# Supplementary material for: Study protocol: Local labour market programs–institutional structures, organizational forms and lived experiences
Source: PLoS One. 2024 Jan 24;19(1):e0296242. doi: 10.1371/journal.pone.0296242 (PMC10807781; doi:10.1371/journal.pone.0296242)
Supplement: Attachment — Submitted filename: Responses to reviewers.docx [file pone.0296242.s001.docx]

**Responses to reviews**

Journal Requirements:
When submitting your revision, we need you to address these additional requirements.

**1. Please ensure that your manuscript meets PLOS ONE's style requirements, including those for file naming. The PLOS ONE style templates can be found at**

[https://journals.plos.org/plosone/s/file?id=wjVg/PLOSOne_formatting_sample_main_body.pdf](https://journals.plos.org/plosone/s/file?id=wjVg/PLOSOne_formatting_sample_main_body.pdf) and

[https://journals.plos.org/plosone/s/file?id=ba62/PLOSOne_formatting_sample_title_authors_affiliations.pdf](https://journals.plos.org/plosone/s/file?id=ba62/PLOSOne_formatting_sample_title_authors_affiliations.pdf)

**2. Thank you for stating the following financial disclosure:**

"Funded studies FORTE, Grant number: STY-2021/0005 [forte.se/eng](https://forte.se/eng)"

Please state what role the funders took in the study. If the funders had no role, please state: "The funders had no role in study design, data collection and analysis, decision to publish, or preparation of the manuscript."

Please include this amended Role of Funder statement in your cover letter; we will change the online submission form on your behalf.

**Answer to Editor:**

The role of Funder is now included in the cover letter: "The funders had no role in study design, data collection and analysis, decision to publish, or preparation of the manuscript."

**3. In your Data Availability statement, you have not specified where the** minimal data set underlying the results described in your manuscript can be found.

See answer below (4)

**4. If you wish to make changes to your Data Availability statement, please describe these changes in your cover letter and we will** **update your Data Availability statement to reflect the information you provide.**

**Answer to Editor:**

The paragraph below is now also included in the cover letter

Since this manuscript is a protocol article we do not yet have any data from either interviews or surveys. The data collection will start later this year.
We have established a plan for data handling which means that all data collected will be stored in the university's secure system until any request to access the data.
We follow the ethical guidelines from the ethical review board, which also govern which data can be made available. We strive to make all data available upon request.

**5. Please review your reference list to ensure that it is complete and correct.** If you have cited papers that have been retracted, please include the rationale for doing so in the manuscript text, or remove these references and replace them with relevant current references. Any changes to the reference list should be mentioned in the rebuttal letter that accompanies your revised manuscript. If you need to cite a retracted article, indicate the article’s retracted status in the References list and also include a citation and full reference for the retraction notice.

**Answer to Editor:**

See answer to reviewer below (1).

**Additional Editor Comments:**
This is a fine work and it could be publishable. However, the literature is not conving without having the comprehensive synthesis of labour market and market evaluation. **Please refer to these two studies** (encourage) to enhance your arguments:

[a] Litwok, D. (2022). Estimating the Impact of Emergency Assistance on Educational Progress for Low-Income Adults: Experimental and Nonexperimental Evidence. Evaluation Review, 0(0). [https://doi.org/10.1177/0193841X221118454](https://doi.org/10.1177/0193841X221118454)

[b] Caliendo, M., & Tübbicke, S. (2022). Do Start-Up Subsidies for the Unemployed Affect Participants’ Well-Being? A Rigorous Look at (Un-)Intended Consequences of Labor Market Policies. Evaluation Review, 46(5), 517–554. [https://doi.org/10.1177/0193841X20927237](https://doi.org/10.1177/0193841X20927237)

**Answer to Editor:**
Thank you for suggesting these references. We have now added reference to Litewok (2022 on page 16, and Caliendo & Tübbicke (2022) on page 3 and 14.


**Reviewers' comments:**
Reviewer's Responses to Questions
Comments to the Author

**1.** **The biggest concern I have about this study is that the authors stressed inclusion in their title and introduction.** However, this is not evidenced in the research questions. A large part of the study seems to only focus on evaluate the effectiveness of LLMPs, which are relatively irrelevant to "inclusion" metrics. The authors may either stress less of inclusion in their introduction, or make inclusion their key evaluation metric in the four work packages. It is similar for the COVID exogenous shock, which has appeared in many parts of the protocol, but with limited focus in the research questions.

**Answer to reviewer #1:**

We understand that this might be confusing since we are not studying inclusion per se, but the long-term goal of our research project is for participants in LLMPs to be included in the labor market. We have tried to make explicit that we focus on the inclusion of LLMP participants perspectives in the measurement of LLMPs effectiveness and that they should have the opportunity to receive the support they need in order for them to be included in the labor market.

We have also removed references to the Covid pandemic because it is not the focus of the study. This also means that the reference list has changed in which the following references has been removed:

Juranek, S., et al., *Labor market effects of COVID-19 in Sweden and its neighbors: Evidence from novel administrative data.* NHH Dept. of Business and Management Science Discussion Paper, 2020(2020/8).

Arbetsförmedlingen. *Coronakrisen slår hårt mot äldre arbetstagare*. AV nyheter 2021.

SCB. *Kraftig försämring på arbetsmarknaden för unga och utrikesfödda. Arbetskraftsundersökningen (AKU)* Arbetskraftsundersökningen (AKU) 2020.

**2. While the significance of this study has been justified, as LLMPs broadly** serve as a complement of Swedish state function of implementing labour market policies, the significance is argued at national level. The main comparative study covers two regions of Sweden, but I cannot see the selection of these two cases well justified. Have the authors considered all regions in Sweden to make this choice or is this based on feasibility? I would like to see more evidence, and a similar concern is raised to the choice of municipality sample.

**Answer to reviewer #1:**

Thank you for these comments. You address an important issue on the selection of regions and municipalities. One reason behind the choice of regions was that the two regions would represent regions with significant different conditions in, for example, growth, population development and labor market challenges. This means that one of the regions would be one of the Swedish metropolitan regions and the other regions would be a region with more challenging conditions in unemployment rates, population and growth.

Considering the choices of municipalities in each region we have based the selection on similarities regarding the municipality’s relative position within each region. We have then created four municipal pairs with one municipality from each region.

**3. The research questions are sometimes too broadly defined.** Asking "what" questions is inadequate to construct testable hypotheses. I would like to see more detailed plan of variables to be collected and examined, if possible. For example, I would consider Research Question 7 in Work Package 3 is a more testable set of questions. Some of the rest are relatively a bit ambiguous.

**Answer to reviewer #1:**

Thank you for this comment and we hope that this might be possible for us to consider in following projects. In this program Work Package 3 is more of an explorative study since this is the first study that will investigate the perspective of LLMP participants. The research questions presented for Work Package 3 are the ones developed for the granted project and the questions that have now received ethical permission. This means that we can not change them at this point, and at this point we want to have a more explorative and qualitative approach in Work Package 3.

**4. The authors state this is a longitudinal study.** It would be better if the time coverage is also specified. How will the surveys collect information in the past, and for how long will the study cover?

**Answer to reviewer #1:**We have specified the time coverage of the research program. The program covers six years and information in the past will not be collected although, there are some statistics available at Kolada. Our research will cover the six years period of the project.

**5. On page 26, there is a typo** at the beginning of the risks and challenges section. "ranging from the intitutional lever" which should have been "level".

**Answer to reviewer #1:**

Thank you for noticing this typo. This is now changed according to suggestion.
